# Chromosomal phase improves aneuploidy detection in non-invasive prenatal testing at low fetal DNA fractions

**DOI:** 10.1038/s41598-022-14049-5

**Published:** 2022-07-14

**Authors:** Giulio Genovese, Curtis J. Mello, Po-Ru Loh, Robert E. Handsaker, Seva Kashin, Christopher W. Whelan, Lucy A. Bayer-Zwirello, Steven A. McCarroll

**Affiliations:** 1 Program in Medical and Population Genetics, Broad Institute of MIT and Harvard, Cambridge, MA 02142 USA; 2 Stanley Center for Psychiatric Research, Broad Institute of MIT and Harvard, Cambridge, MA 02142 USA; 3 Department of Genetics, Harvard Medical School, Boston, MA 02115 USA; 4 Division of Genetics, Department of Medicine, Brigham and Women’s Hospital and Harvard Medical School, Boston, MA 02115 USA; 5 Steward St. Elizabeth’s Medical Center, Tufts University School of Medicine, Boston, MA 02135 USA

**Keywords:** Haplotypes, Haplotypes, Genetic testing, Chromosome abnormality, Aneuploidy

## Abstract

Non-invasive prenatal testing (NIPT) to detect fetal aneuploidy by sequencing the cell-free DNA (cfDNA) in maternal plasma is being broadly adopted. To detect fetal aneuploidies from maternal plasma, where fetal DNA is mixed with far-larger amounts of maternal DNA, NIPT requires a minimum fraction of the circulating cfDNA to be of placental origin, a level which is usually attained beginning at 10 weeks gestational age. We present an approach that leverages the arrangement of alleles along homologous chromosomes—also known as chromosomal phase—to make NIPT analyses more conclusive. We validate our approach with in silico simulations, then re-analyze data from a pregnant mother who, due to a fetal DNA fraction of 3.4%, received an inconclusive aneuploidy determination through NIPT. We find that the presence of a trisomy 18 fetus can be conclusively inferred from the patient’s same molecular data when chromosomal phase is incorporated into the analysis. Key to the effectiveness of our approach is the ability of homologous chromosomes to act as natural controls for each other and the ability of chromosomal phase to integrate subtle quantitative signals across very many sequence variants. These results show that chromosomal phase increases the sensitivity of a common laboratory test, an idea that could also advance cfDNA analyses for cancer detection.

## Introduction

Since the discovery of cell-free fetal DNA in maternal plasma in 1997^1^, new technologies based on deep sequencing of the cell-free DNA (cfDNA) have been replacing earlier prenatal screening methods based on ultrasound and serum biochemical assays^2^. Non-invasive prenatal testing (NIPT) is now clinically available beginning at 9–10 weeks gestational age^3–5^ (GA) and poses no risk to the fetus, whereas invasive prenatal diagnostic procedures, such as chorionic villus sampling and amniocentesis, come with the risk of miscarriage and are not available, respectively, before 10 and 15 weeks GA^6^. To date, more than 10 million women have received NIPT^7^. The core analytical challenge in NIPT is that only a small fraction of the DNA in maternal plasma is fetally derived (known as the fetal DNA fraction). Thus, analytical methods must detect deviations from a “normal” fetal genome in the presence of far-larger quantities of maternal DNA. Two kinds of analytical approaches are routinely used to detect fetal aneuploidies from the cfDNA.

*Quantitative methods* use massively parallel shotgun sequencing (MPSS), then count the numbers of sequence fragments arising from a chromosome of interest and compare this with reference chromosome(s). Increased (decreased) counts for a specific chromosome can suggest trisomy (monosomy) of that chromosome in the fetal genome^8,9^. For example, chromosome 21 generates about 1.0% of sequence fragments, a fraction that in principle rises only to 1.05% if 10% of the cfDNA arises from a fetal genome with chromosome 21 trisomy, a difference that must be distinguished from random chance. A further challenge is that, while statistical sampling noise can be addressed by sampling and sequencing larger numbers of sequence fragments, biological and laboratory-process driven sources of variation cannot be addressed by simply sequencing more molecules^10^. Quantitative methods can detect fetal trisomies at fetal DNA fractions as low as 2%^11^, but have reduced sensitivity at fetal DNA fractions lower than 4%^12,13^. As a result, most NIPT analyses based on quantitative methods have a strict 3.5–4.0% fetal DNA fraction threshold for returning a result^4,5,14^.

*Single nucleotide polymorphisms (SNP)-based methods* use targeted sequencing to generate very many reads from thousands of commonly heterozygous SNPs, then count the number of sequence fragments arising from each allele. For each chromosomes these allelic ratios are then assessed for whether they best fit a euploid or an aneuploid scenario^15–17^. In the cfDNA that is of 10% fetal origin, homologous alleles at maternally heterozygous loci, which are present at a 1:1 ratio in purely maternal DNA, could be present at ratios of 11:9, 10:10, or 9:11 (depending on fetal genotype) if mixed with DNA from a euploid fetus and at ratios of 12:9, 11:10, 10:11, or 9:12 in the case of a trisomy fetus. SNP-based methods are robust to sources of variation that affect homologous chromosomes in an equal manner such as most amplification biases^18^. As a result, a commercially available SNP-based test can return results for fetal DNA fractions as low as 2.8%^19^, while maintaining high accuracy rates^20–24^.

Results of NIPT analyses can be inconclusive, especially in the context of aneuploid pregnancies. Owing to low fetal DNA fractions or high assay variance, 3.8% of pregnant women ordering a commercially available SNP-based NIPT^25,26^ receive an inconclusive result at first blood draw. The dealy until a conclusive aneuploidy determination is received can cause anxiety, dissatisfaction and, for aneuploid pregnancies, limit patient choices^27^. In addition, trisomy 13, 18, and digynic (maternal) triploidy pregnancies are associated with lower fetal DNA fractions^25,28–33^, possibly as a result of placental abnormalities or smaller placental sizes^34^. As a result, among pregnant mothers with an aneuploid fetus, the population for whom conclusive results may be most urgent, 10.6% receive an inconclusive result, with trisomy 18 pregnancies having the highest rate at 25%^3,19^.

Here we investigate how chromosomal phase—the arrangement of alleles along homologous chromosomes—can be used to make conclusive NIPT determinations at low fetal DNA fractions using the SNP-based method. While current DNA sequencing technologies can detect an individual’s SNP genotypes, they do not ascertain the arrangement of SNP alleles along homologous chromosomes (homologs). Experimental methods to infer homologs at chromosomal scale (such as microfluidic separation of individual chromosomes) do exist^35–38^, but are labor-intensive and expensive and have not been applied in clinical settings. A scalable computational approach to chromosomal phasing (“statistical phasing”) draws upon available genotype data from large population cohorts, utilizing the fact that at any genomic locus a proband is likely to share, with some individuals in any cohort, long DNA segments inherited from (unknown) distant relatives^39–41^ which can then be phased by comparing the genotypes. Statistical phasing is highly scalable but accurate only at megabase (rather than whole-chromosome) scales when using publicly available haplotype reference panels^42^. As a result, the two inferred homologs will often include “switch errors” at unknown sites which can be thought of as pseudo recombination events between the two true homologs. Alternatively, genotypes from direct relatives can provide accurate estimates of the arrangement of alleles along homologous chromosomes, but cannot be as easily retrieved in routine clinical contexts. We show how combining previously developed frameworks for inferring fetal genotypes from allelic read counts^43–45^ with an analytical framework expanding on one we developed to detect large mosaic chromosomal alterations in blood-derived DNA at low cell fractions (as low as 1%)^46–48^, we can improve fetal ploidy inference. We investigate the effectiveness of this approach via in silico simulations, then validate it by analyzing allelic read counts generated from the plasma of a pregnant mother carrying a trisomy 18 fetus.

## Results

**Figure 1:**
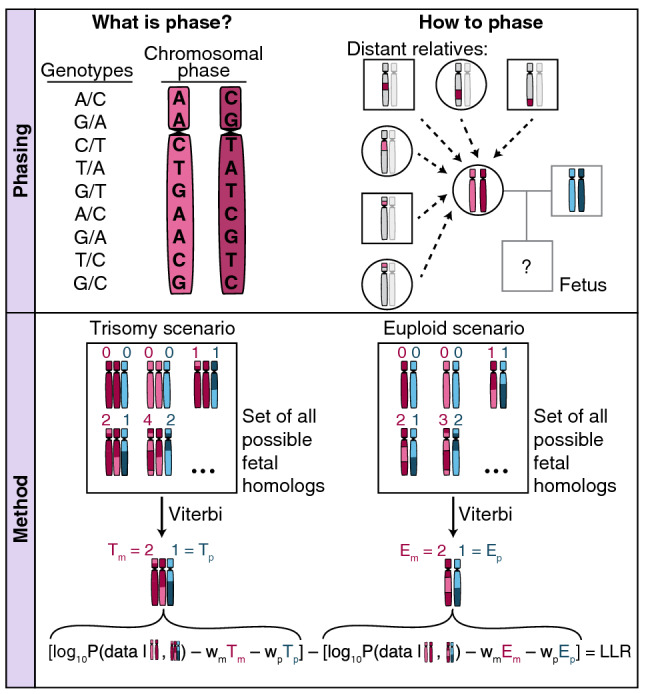
Schematic representation of the role of chromosomal phase in the computation of the discrimination statistic Graphical representation of how chromosomal phase is inferred through relatives on the top and how the space of possible fetal inheritance configurations is explored on the bottom to identify, through the Viterbi algorithm, the two most likely configurations for both the trisomy and the euploid scenario. A log_10_ likelihood ratio (LLR) discrimination statistic based on the likelihood of the data and weighed by the number of crossovers and phase switch errors needed to explain the fetal genome is then computed. The weight applied to the number of crossovers and switch errors is akin to assigning a prior over the space of possible fetal inheritance configurations that concentrates the probability towards configurations that can be explained with fewer switches starting from parental homologs.

At a technical level, the approach in our NIPT analysis framework is to first infer chromosomal phase for the assayed SNPs from a pregnant woman’s genome, then determine the most likely sequence of fetal inheritance states for the fetal maternal homologs among those compatible with an aneuploid scenario as well as the most likely sequence for a euploid scenario, and then score these two as explained below. To efficiently explore the space of all possible sequences, we frame the allelic read counts as the observed states of a Hidden Markov Model with the hidden states corresponding to the inheritance states for both fetal maternal and paternal homologs and transitions across states corresponding to either switch errors, crossovers, or lack thereof. We then use the Viterbi decoding algorithm to find for each of the aneuploid and the euploid scenarios, the most likely sequence of fetal inheritance states that explains the observed allelic read counts. To account for switch errors in the inferred maternal homologs, and to model crossovers in the maternal meiosis, our model incurs a “penalty cost” for switching across inheritance states. We finally compute a single log_10_ likelihood ratio (LLR) discrimination statistic for the two most likely sequences (Fig. 1) computing likelihoods for the allelic read counts at each SNP (Fig. S1). Strongly negative values of the LLR discrimination statistic indicate a euploid fetal genome (for the chromosome in question), while strongly positive values indicate aneuploidy. For comparative purposes, we also run a simpler less powerful model that does not use chromosomal phase (Methods). We caution that, as the SNP-based method relies on sites heterozygous in the mother to detect aneuploidy of maternal origin, in some rare cases high levels of homozygosity, possibly due to consanguinity or segmental uniparental disomy, could make the data uninformative.

### In silico simulation

Since chromosomal phase inferred through statistical phasing is accurate at only megabase scales, with switch errors at unknown sites along each chromosome, it was essential to evaluate our method under various levels of accuracy for the chromosomal phase, we performed simulations that incorporate various (known) numbers of switch errors. We simulated allelic read counts over a single chromosome as if derived from the cfDNA in the plasma of a pregnant woman with a singleton gestation. To simulate a real case scenario presented by a commercially available SNP-based test^19^, our simulation included 1500 heterozygous loci in the mother’s genome with a sampling of an average of 2000 sequence fragments per locus. At each locus, the expected number of sequence fragments for each allele was estimated from the given fetal DNA fraction and the simulated genotypes with overdispersion parameters drawn from empirical data (“Methods”).

Aneuploidies of maternal origin and are more common than paternal ones^49^ and require higher fetal DNA fractions to detect by SNP-based methods^50^ since the contaminating (majority) of the cfDNA comes from the maternal genome. We thus focused on this most-challenging problem of inferring fetal maternal homologs. In practice, paternal alleles not present in the mother’s genome are readily recognized at loci where the maternal genotype is homozygous^43^, and from these alleles imputing the remaining paternal alleles works well in genomic regions where the paternal homolog harbors a sufficient number of paternal-specific alleles and is unambiguously associated with one haplotype from the reference panel^44^. To focus on the more challenging and common scenario of trisomies of maternal origin we simulated only the fetal inheritance of the maternal homolog and we modeled the paternal homolog as contributing equally towards either allele at maternal heterozygous sites (“Methods”).

**Figure 2:**
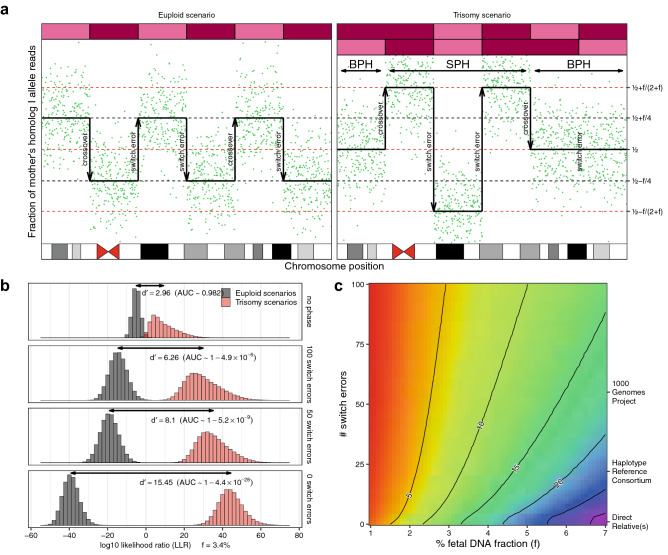
Simulation of log_10_ likelihood ratio (LLR) discrimination statistics. (**a**) Simulated allelic fractions from the maternal plasma of a pregnant woman with crossovers and switch errors. The top bars represent the simulated maternal homologs inherited by the fetus, with magenta and red representing, respectively, mother’s homolog I and mother’s homolog II. Notice that, in the trisomy scenario, switch errors don’t change the expected proportion of alleles when occurring in BPH segments. (**b**) Simulated log_10_ likelihood ratio (LLR) discrimination statistics from simulations with a fetal DNA fraction specified as f = 3.4%, and an average sampling of 2000 sequence fragments at 1500 loci heterozygous for the mother, for both trisomy and euploid scenarios. Sensitivity index d’ between the LLR discrimination statistics for the two models is displayed together with the AUC estimated as if the two LLR discrimination statistics were normally distributed. As the number of switch errors decreases, the ability of the LLR discrimination statistic to distinguish the euploid and trisomy scenarios increases. (**c**) Sensitivity index d’ between LLR discrimination statistics for allelic read counts simulated by trisomy scenarios and those simulated by euploid scenarios as a function of fetal DNA fraction and the number of switch errors. For reference, a representative chromosomal phase accuracy using different chromosomal phasing approaches is reported on the right. Contour lines follow parameters sets with the same sensitivity index d’, indicating scenarios with approximately equivalent power to distinguish euploid and trisomy scenarios.

In the maternal trisomy scenario, the maternal homologous DNA segments can either be the same sequence because they arise from the same homolog (which we refer to as single parental homolog [SPH] segments) or arise from the mother’s two different homologs (which we refer to as both parental homologs [BPH] segments)^51^. Different autosomes have different rates of SPH and BPH segments when presenting as aneuploid. As an example, chromosome 21 trisomies of maternal origin, the most common cause of viable fetal aneuploidies, involve BPH segments far more often than SPH segments^52^. To simulate a scenario that exemplifies the full complexity of the model, we focus on trisomies with two outer BPH segments and one central SPH segment in the trisomy scenario (Fig. 2a). The code used for the simulation is publicly available.

Using the 1000 Genomes Project haplotype reference panel^53^ or the Haplotype Reference Consortium panel^54^, statistical phasing on average yields, respectively, one switch error every megabase pair or one switch error every 2.5 megabase pairs^42^. A more accurate approach would be to draw upon data from a mother’s direct relative. As an example, chromosomal phasing of maternal genotypes from a previous child’s genotypes, possibly inferred through a previous NIPT analysis, would allow the resolution of the maternal genotypes into transmitted and untransmitted homologs, with only a few switch errors at the locations of the crossovers for the previous child. To address these different plausible scenarios, we ran multiple simulations with variable numbers of switch errors.

We found that chromosomal phase inference provided a dramatic improvement in the ability to distinguish a trisomy fetus from a euploid fetus based on the LLR discrimination statistic (Fig. 2b). This improvement was observed across a wide range of fetal DNA fractions and switch error rates (Fig. 2c). While we should expect additional sources of variation beyond sampling noise to affect the allelic counts in a real-world test, the simulation validates the intuition that detecting fetal aneuploidy from allelic imbalances in maternal plasma can strongly benefit from knowledge of the chromosomal phase accurate at the scale of megabase pairs, even without necessarily having access to genotypes from direct relatives. We have previously observed a similar increase in detection power in detecting large mosaic chromosomal alterations (for example, due to clonal hematopoiesis) from allelic imbalances at heterozygous loci^46–48^.

We next evaluated this idea using clinical NIPT data from a SNP-based targeted sequencing assay.

### A case study

**Figure 3:**
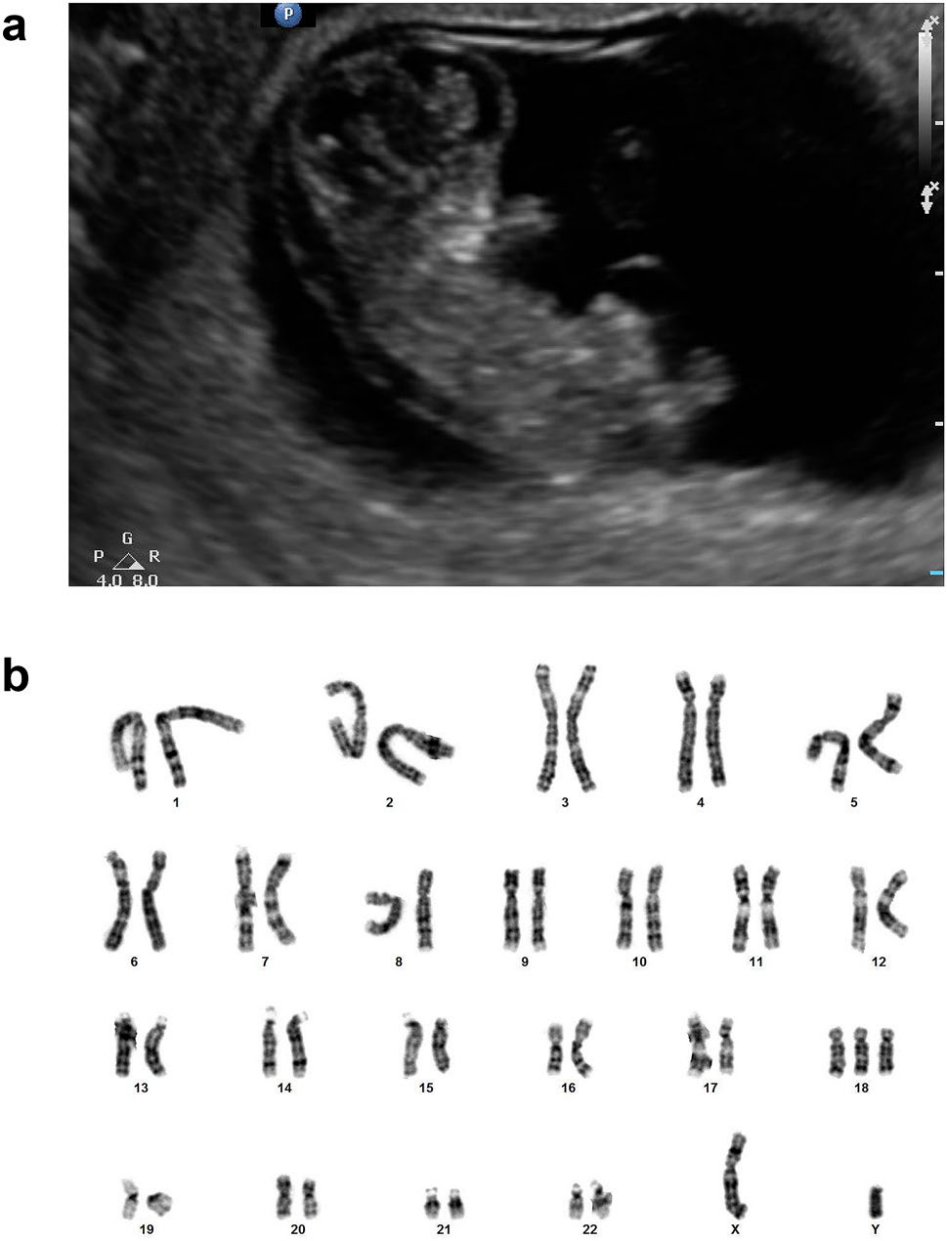
Fetal ultrasound and karyotype. (**a**) Ultrasound image of a trisomy 18 fetus at 11 weeks GA showing potential neck thickening and a crown-rump length of 33 mm. (**b**) Fetal karyotype. Abnormal 47,XY, + 18 male chromosome karyotype with an extra chromosome 18 observed in mitotic cells obtained from examination of products of conception.

We turned to the analysis of a case study involving a nulliparous 35-year-old South Asian woman with a singleton gestation. The mother received an abnormal ultrasound finding at 11 weeks GA, prompting care providers to pursue NIPT through a commercially available SNP-based test. The test returned 10 days later as inconclusive. The fetal DNA fraction was estimated as f = 3.4%. However, the test reported an increased risk (of 5.7%) for digynic triploidy, trisomy 18, or trisomy 13, solely based on the low fetal DNA fraction (given maternal age, GA, and a maternal weight of 145 pounds) which is associated with increased risk of adverse outcome^25,33^. The level of hCG in blood was measured at 32,378. The ultrasound examination at 11 weeks GA revealed potential neck thickening possibly due to a small cystic hygroma formation and a crown-rump length of 33 mm consistent with a 10 week GA size fetus and therefore compatible with intrauterine growth restriction (Fig. 3a).

**Figure 4:**
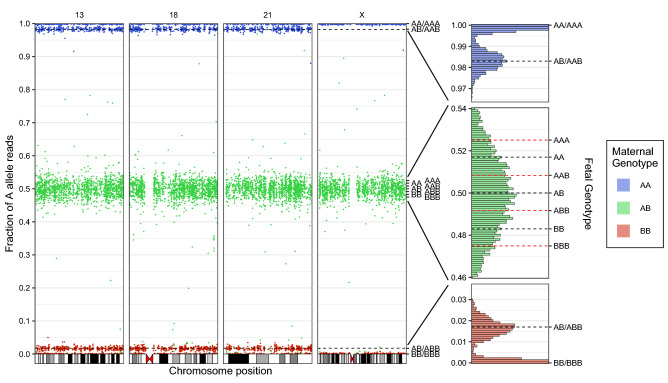
SNP-based targeted sequencing data without chromosomal phase. Graphical representation of sequencing data from the SNP-based targeted sequencing of maternal plasma obtained from the pregnant mother at 11 weeks GA with a fetal DNA fraction estimated as f = 3.4%. Points correspond to the fraction of alternate (A) allele read counts as a fraction of the overall number of reads at SNP loci sampled by more than 200 sequence fragments. At loci for which the mother is homozygous, the fetal genotype can be inferred with high accuracy. Black and red dotted lines represent the expected fractions of A alleles for different combinations of maternal and fetal genotypes in the case of, respectively, a euploid and trisomic fetus. The lack of SNP loci at fractions for which the mother is expected to be homozygous and the fetus is expected to be heterozygous along the chromosome X homologs is indicative of a male fetus.

Sequence data from the SNP-based test were provided by the test provider. These data included 29.8 million targeted sequence reads interrogating approximately 3000 common SNPs for each of chromosomes 13, 18, 21, and X. SNPs sampled by more than 200 sequence fragments were on average sampled 2000 times. We used a maximum likelihood-based approach that modeled both the maternal and the paternal inheritance states (Methods and Fig. S1). We used knowledge of both maternal and paternal genotypes inferred through high coverage MPSS (Methods). We notice that paternal genotypes are often not available in a clinical setting, but our framework could be easily extended to modeling the fetal paternal haplotype without using knowledge of paternal genotypes^44^. Analysis of the allelic fractions without using chromosomal phase identified a male fetus with no trisomies of paternal origin over chromosomes 13, 18, 21, and X (Fig. 4). However, due to the low fetal DNA fraction, analysis of trisomies of maternal origin (without using chromosomal phase) was inconclusive: the LLR discrimination statistics computed from maternal genotypes alone were − 7.67, 1.13, − 5.10 for, respectively, chromosomes 13, 18, and 21 (Table 1), similar to what was expected from simulated data (Fig. 2b) and therefore insufficient to make a conclusive aneuploidy determination.

**Table 1:** Fetal DNA fraction estimates and discrimination statistics across autosomes.

Autosome	SNP-based targeted sequencing (11 weeks GA)							Massively parallel shotgun sequencing (15 weeks GA)						
	# Opposite homozygote sites	Average # of sequence fragments	Estimated fetal DNA fraction	# Maternal heterozygous sites	LLR discrimination statistic			# Opposite homozygote sites	Average # of sequence fragments	Estimated fetal DNA fraction	# Maternal heterozygous sites	LLR discrimination statistic		
					No phase	Phase with 1000 Genomes Project	Phase with direct relatives					No phase	Phase with 1000 Genomes Project	Phase with direct relatives
1	–	–	–	–	–	–	–	20,920	8.23	5.84%	115,058	4.15	− 58.28	− 119.15
2	–	–	–	–	–	–	–	20,640	8.63	6.02%	120,127	4.14	− 58.48	− 135.13
3	–	–	–	–	–	–	–	19,614	8.74	5.96%	105,013	1.46	− 51.67	− 114.55
4	–	–	–	–	–	–	–	16,645	9.05	5.88%	108,072	9.96	− 59.59	− 132.10
5	–	–	–	–	–	–	–	17,653	8.75	5.94%	88,874	− 3.10	− 49.56	− 103.84
6	–	–	–	–	–	–	–	18,613	8.70	5.91%	91,153	5.54	− 60.11	− 95.12
7	–	–	–	–	–	–	–	14,425	8.43	6.04%	79,925	7.34	− 41.72	− 70.86
8	–	–	–	–	–	–	–	12,923	8.61	6.20%	79,248	− 5.49	− 56.19	− 106.93
9	–	–	–	–	–	–	–	10,206	8.37	5.92%	61,287	− 0.14	− 33.72	− 65.76
10	–	–	–	–	–	–	–	13,520	8.22	5.97%	72,521	2.60	− 21.68	− 68.18
11	–	–	–	–	–	–	–	11,777	8.36	6.01%	68,641	− 3.65	− 34.85	− 79.84
12	–	–	–	–	–	–	–	12,755	8.41	5.96%	65,875	3.61	− 39.32	− 74.47
13	354	1930	3.46%	1375	− 7.68	− 12.00	− 36.01	10,276	8.92	6.04%	52,609	1.94	− 24.45	− 49.46
14	–	–	–	–	–	–	–	9470	8.45	5.87%	47,726	13.21	− 11.84	− 42.37
15	–	–	–	–	–	–	–	6713	8.12	5.65%	40,571	4.44	− 6.80	− 34.05
16	–	–	–	–	–	–	–	8652	7.27	6.11%	41,622	2.44	− 22.13	− 17.27
17	–	–	–	–	–	–	–	5978	7.02	6.04%	36,397	− 0.79	− 12.57	− 35.71
18	384	1975	3.29%	1,11	1.13	14.94	26.33	6684	8.92	5.64%	41,585	2.60	26.83	39.84
19	–	–	–	–	–	–	–	5491	6.12	6.23%	29,491	− 1.21	− 7.47	− 30.66
20	–	–	–	–	–	–	–	5840	7.51	5.62%	30,894	2.78	− 2.28	− 23.85
21	362	1914	3.36%	1,30	− 5.10	− 14.48	− 33.77	3762	8.33	5.89%	19,878	0.00	− 9.26	− 16.52
22	–	–	–	–	–	–	–	3699	6.50	5.75%	18,235	2.39	− 6.99	− 9.64

SNP-based analyses results for the targeted and MPSS data generated from the cfDNA of the plasma obtained from the pregnant mother. For each autosome we report (i) the number of covered SNPs for which the parents’ genotypes are opposite homozygotes, (ii) the average number of sequence fragments overlapping the SNPs, (iii) the estimated fetal fraction from the opposite homozygotes SNPs, (iv) the number of covered SNPs for which the mother is heterozygous, and (v) the LRR discrimination statistics for the model without chromosomal phase, with chromosomal phase inferred from 1000 Genomes Project haplotype reference panel, and with chromosomal phase inferred from direct relatives. Targeted data only covers autosomes 13, 18, 21.

We then re-analyzed the same NIPT data combining it with knowledge of chromosomal phase for the parental genotypes inferred from the 1000 Genomes Project haplotype reference panel (“Methods”). The analysis yielded a highly conclusive determination of trisomy 18, with an LLR discrimination statistic of 14.64 for chromosome 18 and LLR discrimination statistics of –12.00 and –14.48 for, respectively, chromosomes 13 and 21.

**Figure 5:**
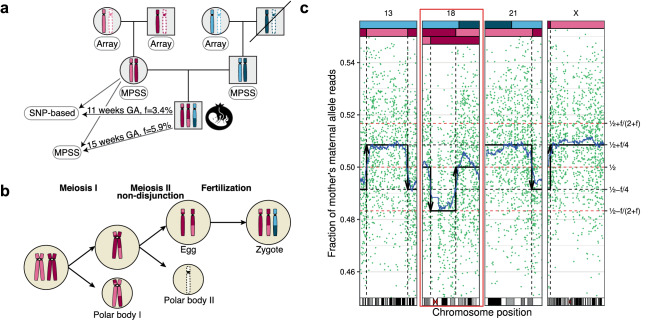
SNP-based targeted sequencing data with chromosomal phase. (**a**) Pedigree with parents and grandparents of the trisomy 18 fetus. MPSS data of DNA from saliva were available for the parents and microarray genotype data of DNA from saliva were available for the grandparents. Data from the SNP-based targeted sequencing and MPSS of maternal plasma using the Illumina Nextseq 500 platform were available at, respectively, 11 weeks and 15 weeks GA. (**b**) Schematic representation of the formation of a trisomic zygote through missegregation of chromosomes during maternal meiosis II. (**c**) Graphical representation of sequence data at loci heterozygous for the mother from the SNP-based targeted sequencing of maternal plasma at 11 weeks GA with a fetal DNA fraction estimated as f = 3.4%. Each green point corresponds to the fraction of the mother’s maternal allele reads at any of the 13,926 SNP loci that are consistent with heterozygous genotypes for the mother and were covered by more than 200 sequence fragments. The two black dotted lines represent the expected fractions of the mother’s maternal alleles in the case of a euploid fetus, and the three red dotted lines represent the expected fractions in the case of a fetus with trisomy of maternal origin. The blue line is a centered rolling mean across 200 consecutive heterozygous SNPs. The top bars represent the inferred inherited homologs of the fetus, with magenta, red, cyan, and blue colors representing, respectively, mother’s maternal, mother’s paternal, father’s maternal, and father’s paternal homologs. Chromosome 18, with three fetal homologs inferred, is highlighted in red. It is important to note that the algorithm to infer the inherited homolog segments takes also into account information about the homologs transmitted from the father of the fetus and allelic fractions at SNP loci consistent with homozygous genotype for the mother and which are not displayed in this figure and that the paternal homologs of the fetus further adds to the sampling noise at loci heterozygous for the mother.

The statistical phasing that enabled this conclusive analysis is expected to be accurate only at megabase scales. To evaluate the effect of phase switch errors, we compared to analyses in which we used genotypes from direct relatives (Fig. 5a) to infer the actual maternal homologs. We identified 5,355 switch errors across 198,684 autosomal loci heterozygous for the mother and present in microarray genotype data available for her parents, corresponding to an average of one switch error every 0.5 megabase pairs.

Further enhancing chromosomal phase using genotypes from direct relatives to resolve switch errors in the inferred maternal and paternal homologs enabled an even more conclusive aneuploidy determination, with the same analysis yielding an LLR discrimination statistic of 26.33 for chromosome 18, and LLR discrimination statistics of − 36.01 and − 33.77 for, respectively, chromosomes 13 and 21 (Table 1), similar to what was expected from simulated data (Fig. 2b). The inheritance states with the highest likelihood for chromosome 18 consisted of three segments: two distal BPH segments and one central SPH segment spanning the centromere. This suggests a chromosomal non-disjunction in maternal meiosis II with one crossover on each chromosome arm, the most common type of maternal chromosome 18 non-disjunction^55^ (Fig. 5b,c).

### Validation with massively parallel shotgun sequencing data

To investigate whether the same analytical framework could be applied to MPSS data (i.e., without targeting SNPs), we first simulated MPSS data at a number of heterozygous loci in the mother’s genome compatible with an empirical scenario with a sampling of an average of either 10, 20, or 30 sequence fragments per locus (Fig. S2). These simulations indicated that comparable power to the SNP-based targeted approach could be achieved by sampling 10–20 sequence fragments per locus to detect trisomy 13 and 18 and a little more than 30 sequence fragments to detect trisomy 21. The same framework indicates that at the simulated sampling averages a non-targeted approach would likely be unable to reliably detect the presence of the 22q11.2 microdeletion at low fetal DNA fractions (Fig. S3), similar to how commercially available SNP-based NIPT requires a minimum fetal DNA fraction of 6.5% for the detection of the 22q11.2 microdeletion of maternal origin^56^.

We then generated a sequencing library from the cfDNA obtained from maternal plasma of the same pregnant mother at 15 weeks GA, from which we sampled an average of 8.4 sequence fragments per polymorphic locus without targeting SNPs (Methods). According to simulations, this level of sampling was likely sufficient to validate the results from the SNP-based targeted approach if inferring chromosomal phase using genotypes from direct relatives at an f = 3.4% fetal DNA fraction (Fig. S3). However, and not surprisingly, the measured fetal DNA fraction in maternal plasma at 15 weeks GA was estimated to be f = 5.9%, higher than the one measured at 11 weeks GA, reflecting the later GA. Similar to the analysis of the SNP-based targeted sequencing data, the LLR discrimination statistics computed from maternal genotype alone were insufficient to make a conclusive aneuploidy determination (Table 1). By inferring the parental homologs using the 1000 Genomes Project haplotype reference panel^53^, the LLR discrimination statistic for chromosome 18 was 26.83, while it was –24.45 and –9.26 for, respectively, chromosome 13 and chromosome 21, and the highest value across the remaining autosomes was − 2.28. Further improving the chromosomal phase using direct relatives of the parents, the same analysis yielded an LLR discrimination statistic of 39.84 for chromosome 18 and –49.46 and –16.52 for, respectively, chromosome 13 and chromosome 21, similar to what was expected from simulated data (Fig. S3), while the highest value across the remaining autosomes was –9.64 (Table 1). Although either phasing strategy produced positive values for chromosome 18 and negative values for all the other autosomes, leading to the same ploidy conclusions, it did more confidently so when genotypes were phased using direct relatives in agreement with our simulations (Fig. 2c). The inheritance states with the highest likelihood across all chromosomes included 52 crossovers in the maternal meiosis and 20 crossovers in the paternal meiosis (Fig. S4), in agreement with measured recombination-rate differences between the sexes^57^, although it is likely that crossovers near the telomeres were under-ascertained in our analysis. Crossover localizations along chromosomes 13, 18, 21, and X were highly consistent between the SNP-based targeted and the SNP-based MPSS analyses.

### Confirmation of aneuploidy by histopathology and cytogenetic analysis

Subsequent histopathological examination of products of conception revealed chronic villi with basement membrane calcifications, consistent with histomorphological findings in trisomic pregnancies^58^. Cytogenetic analysis through in situ tissue culture for Giemsa-banded chromosome analysis confirmed the presence of an extra chromosome 18 in 20 mitotic cells in 5 cultures yielding a final non-mosaic 47,XY, + 18 diagnosis for the fetus (Fig. 3b).

## Discussion

NIPT is transforming the ascertainment of genetically complicated pregnancies, but its application has been limited to pregnancies in which the fetus makes a sufficient contribution to the cfDNA in maternal plasma. Here we investigated the possibility that a different way of analyzing the data, drawing upon available population-level DNA data or genotypes from direct relatives to infer chromosomal phase, could make such analyses more conclusive.

We showed through simulations that chromosomal phase inferred through statistical phasing using publicly available haplotype reference panels can be used to enhance SNP-based NIPT by lowering the fetal DNA fraction limit of detection for aneuploidy. Increasing the accuracy of chromosomal phase to the scale of tens of megabase pairs can make such determination even more conclusive. We then presented an empirical case in which a definitive determination of fetal trisomy 18 could be achieved once the allelic resolution of maternal homologs was used to reinterpret the SNP-based targeted sequencing data generated from a maternal plasma sample with a fetal DNA fraction estimated as f = 3.4%. We further showed that the same framework can be used to enhance the interpretation of MPSS data generated from a maternal plasma sample from the same pregnancy with a fetal DNA fraction estimated as f = 5.9% with an average of 8.4 sampled sequence fragments per polymorphic locus. The data from both tests are available in the public domain through the Personal Genome Project^59^.

While the only commercially available SNP-based NIPT that we are aware of at the time of this writing does use chromosomal phase to make the aneuploidy determinations^60–65^, it is not clear to what extent. Most Further debates comparing quantitative methods to SNP-based methods^64–67^ will need to account for the value of chromosomal phase in decreasing the fetal DNA fraction limit of detection for SNP-based methods. Of course, only large-scale clinical trials based on empirical data obtained in real-world clinical contexts can fully evaluate the improved resolution of this approach at low fetal DNA fractions, as the strength of SNP-based methods in determining fetal aneuploidy lies the resilience by design to sources of variation that affect homologs in an equal manner and which might be difficult to model through simulations. We also caution that although SNP-based methods do not need to model homologs-neutral amplification biases, they would still benefit from modeling biological processes affecting maternal and fetal DNA molecules in different ways across the chromosomes, such as DNA molecule sizes^43^ and preferred DNA ends^68^, whose effects are insufficiently characterized to model in our simulations. Similarly, experimental strategies to enrich fetal DNA can also be combined with the method we have proposed.

While in the case study presented here we obtained chromosomal phase to the scale of tens of megabase pairs through the use of direct relatives, alternative strategies are feasible. For mothers who have already received the same NIPT for a previous pregnancy, a provider of SNP-based NIPT could infer maternal homologs at a scale limited only by the crossover events in the previous conception, provided that a sufficient fetal DNA fraction was achieved in the previous test, similar to how previous conceptions have been used to infer parental homologs for the determination of transmission of single-gene recessive mutations in future pregnancies^69^. For pregnant mothers receiving the test for the first time, a provider could attempt to infer chromosomal phase through detecting shared DNA segments with distant relatives identified among mothers and fetuses whose homologs have already been successfully resolved in previous tests. Indeed a current SNP-based NIPT provider now claims to leverage information from data generated about the previous 1.6 million sequenced mothers for the purpose of better resolving the homologs^24^ which shows that chromosomal phasing could be retrieved algorithmically with *negligible* computational costs.

Since recombination is infrequent along human chromosomes, even distantly related individuals tend to share DNA segments tens of megabases long and these matches can be used to resolve the chromosomal phase of the underlying DNA segments^39–41^. As an example, an empirical analysis showed that chromosome 12 homologs can be resolved in individuals from the UK Biobank, a cohort of 500,000 volunteers from the United Kingdom, with an average of just 1.6 switch errors, equivalent to one switch error every 60 megabase pairs^47^. Although the UK biobank cohort was analyzed using a DNA microarray assaying approximately 22 k, 19 k, and 10 k markers for, respectively, chromosomes 13, 18, and 21, this is not too dissimilar from the targeted approach presented here, where approximately 3 k markers were assayed for each of those three chromosomes. A SNP-based NIPT provider without access to its own large pool of previously analyzed samples could harness available genotype data from biobank cohorts to infer chromosomal phasing using freely available software^42^.

The ability to determine fetal aneuploidy at lower fetal DNA fractions could benefit cases in which an inconclusive result would otherwise be returned, possibly at an early stage of the pregnancy when it would cause stress and anxiety and limit patient choices while waiting for a redraw to be processed or amniocentesis to become a viable option^27^. Chromosomal phase could also provide increased power to detect tumors harboring large mosaic gains, losses, and copy-neutral loss of heterozygosity of genomic segments from the sequencing of the cfDNA from plasma^62^ which also contains tumor DNA at low cell fractions. We have shown it possible to infer aneuploidy in blood-derived DNA at cell fractions as low as 1%^46–48^, and similar efforts with the cfDNA from plasma could strengthen nascent efforts toward early cancer detection and monitoring of cancer relapse^70^.

Our results are a reminder that NIPT for aneuploidy is a test of hypotheses about chromosomes in the fetus and that chromosomes are inherited from one generation to the next as blocks that are tens of megabase pairs long. Knowledge of the arrangement of alleles along homologous chromosomes can have an important role, sometimes in unexpected ways.

## Methods

### NIPT simulation

To simulate allelic read counts along a chromosome, we first simulate the inheritance states sequence T = (t_1_, …, t_1500_) of maternal inheritance for the fetus. We assume alleles at each heterozygous locus are assigned to homolog I and homolog II through a given chromosomal phasing method with s switch errors. In the euploid scenario t_i_, for i ∈ {1, …, 1500}, can assume only two possible values, indicating whether the mother’s homolog I allele (H_1_) or the mother’s homolog II allele (H_2_) is inherited by the fetus, while in the trisomy scenario it can assume three possible values, two SPH states when either the mother’s homolog I allele (H_11_) or the mother’s homolog II allele (H_22_) is inherited twice and one BPH state when both homologs are inherited by the fetus (H_12_). In simulating the inheritance of maternal homologs by the fetus, we restrict our simulation to the case of exactly c = 2 crossovers with two outer BPH segments and one central SPH segment in the trisomy scenario. In the euploid scenario, each crossover and each switch error correspond to changes from inheritance state H_1_ to H_2_ (or H_2_ to H_1_). In the trisomy scenario, each crossover corresponds to a change from inheritance state H_11_/H_22_ to inheritance state H_12_ (or H_12_ to H_11_/H_22_) and each switch error corresponds to a change from inheritance state H_11_ to H_22_ (or H_22_ to H_11_) or no change if the current inheritance state is H_12_.

For a given inheritance state t_i_ and fetal DNA fraction f, we estimate π_i_, for i ∈ {1, …, 1500}, as the fraction of homolog I alleles expected in maternal plasma at loci heterozygous in the mother assuming that the fetus had inherited a paternal homolog contributing half to homolog I and half to homolog II. Given the maternal contribution per allele proportional to 1-f and the fetal contribution per allele proportional to f (Fig. S1), the fractions are estimated as:

$${\pi }_{i}=\left\{\begin{array}{ccccc}\frac{\left(2+1/2\right)f+\left(1-f\right)}{3f+2\left(1-f\right)}& =& 1/2+f/\left(2+f\right)& if& {t}_{i}={H}_{11}\\ \frac{\left(1+1/2\right)f+\left(1-f\right)}{2f+2\left(1-f\right)}& =& 1/2+f/4& if& {t}_{i}={H}_{1}\\ \frac{\left(1+1/2\right)f+\left(1-f\right)}{3f+2\left(1-f\right)}& =& 1/2& if& {t}_{i}={H}_{12}\\ \frac{1/2f+\left(1-f\right)}{2f+2\left(1-f\right)}& =& 1/2-f/4& if& {t}_{i}={H}_{2}\\ \frac{1/2f+\left(1-f\right)}{3f+2\left(1-f\right)}& =& 1/2-f/\left(2+f\right)& if& {t}_{i}={H}_{22}\end{array}\right.$$

We then simulate allelic read counts sequences A = (a_1_, …, a_1500_) and B = (b_1_, …, b_1500_) such that a_i_ + b_i_ is a negative-binomial random variable with mean 2,000 and overdispersion α = 0.07, irrespective of the inheritance state t_i_, and a_i_ is a beta-binomial random variable with a_i_ + b_i_ trials, expected fraction of successful trials π_i_, and intraclass correlation ρ = 0.001, for i ∈ {1, …, 1500} (Fig. 2a).

Say Ω_3_ is the set of all possible inheritance states sequences X = (x_1_, …, x_1500_) in the trisomy scenario, and Ω_2_ is the set of all possible inheritance states sequences X = (x_1_, …, x_1500_) for the euploid scenario, including inheritance states sequences comprising of any number of crossovers and switch errors. For each simulation of allelic read counts A and B, we compute the log_10_ likelihood ratio (LLR) discrimination statistic:

$$LLR\left(A,B,f,c,s\right)={log}_{10}\left[\frac{{max}_{X\in {\Omega }_{3}}\mathcal{L}\left(X|A,B,f,c,s\right)}{{max}_{X\in {\Omega }_{2}}\mathcal{L}\left(X|A,B,f,c,s\right)}\right]$$

with the likelihood $\mathscr{L}$ computed as follows:

$$\mathcal{L}\left(X|A,B,f,c,s\right)={\prod }_{i=1}^{1500}P\left(k={a}_{i}|n={a}_{i}+{b}_{i},\pi ={\pi }_{i},\rho =0.001\right){\prod }_{i=2}^{1500}L\left({x}_{i-1},{x}_{i},c,s\right)$$

where P(k | n, π, ρ) is the beta-binomial likelihood for k successful trials out of n trials with an expected fraction of successful trials π and intraclass correlation ρ and the transition likelihood L(x_i-1_, x_i_, c, s) is defined as follows:

$$L\left({x}_{i-1},{x}_{i},c,s\right)=\left\{\begin{array}{ccc}1& if& {x}_{i-1}={x}_{i}\\ \left(c+s\right)/1500& if& \left\{{x}_{i-1},{x}_{i}\right\}=\left\{{H}_{1},{H}_{2}\right\}\\ s/1500& if& \left\{{x}_{i-1},{x}_{i}\right\}=\left\{{H}_{11},{H}_{22}\right\}\\ c/1500& if& \left\{{x}_{i-1},{x}_{i}\right\}=\left\{{H}_{11},{H}_{12}\right\}{\text{or}}\left\{{x}_{i-1},{x}_{i}\right\}=\left\{{H}_{12},{H}_{22}\right\}\end{array}\right.$$

Notice that the transition likelihood is affected by the assumed number c of crossovers and the number s of switch errors. Transition likelihoods L(H_1_, H_2_, c, s) and L(H_11_, H_22_, c, s) can be thought of as “penalty costs” to allow to integrate the signal across consecutive polymorphic loci while still accounting for switch errors. This computational expedient allows leveraging chromosomal phase even when imperfect as is the case when inferred using statistical phasing through large genotyped cohorts^40,41^, similar to what has been done in previous work^71,72^. To identify the inheritance states sequences that best fit the data, we use the Viterbi decoding algorithm. This allows to perform a quick and efficient search across the large space of inheritance states sequences with a complexity linear in the number of transitions, but it does not allow to subset the search among inheritance states sequences with a predefined number of switch errors or crossovers nor does it allow for modeling of biological phenomena such as crossover interference.

Say Ψ_3_(c = 2,s) ⊆ Ω_3_ and Ψ_2_(c = 2,s) ⊆ Ω_2_ are the sets of all possible inheritance states for, respectively, a trisomy and a euploid fetus with exactly c = 2 crossovers and s switch errors. For each pair (f, s) we simulate multiple allelic read counts sequences A, B (Fig. 2b) and the corresponding LLR discrimination statistics and, from these, we estimate means μ_3_ and μ_2_ and variances σ_3_^2^ and σ_2_^2^:

$$\begin{array}{ccccc}{\mu }_{3}\left(f,s\right)& =& {E}_{T\in {\Psi }_{3}\left(c=2,s\right)}\left[LLR\left(A,B,f,c=2,s\right)\right]& & \\ {\sigma }_{3}^{2}\left(f,s\right)& =& {E}_{T\in {\Psi }_{3}\left(c=2,s\right)}\left[LLR{\left(A,B,f,c=2,s\right)}^{2}\right]& -& {\mu }_{3}{\left(f,s\right)}^{2}\\ {\mu }_{2}\left(f,s\right)& =& {E}_{T\in {\Psi }_{2}\left(c=2,s\right)}\left[LLR\left(A,B,f,c=2,s\right)\right]& & \\ {\sigma }_{2}^{2}\left(f,s\right)& =& {E}_{T\in {\Psi }_{2}\left(c=2,s\right)}\left[LLR{\left(A,B,f,c=2,s\right)}^{2}\right]& -& {\mu }_{2}{\left(f,s\right)}^{2}\end{array}$$

We use these estimates to compute the sensitivity index d’(f, s) between the two LLR discrimination statistics:

$$d{^{\prime}}\left(f,s\right)=\frac{{\mu }_{3}\left(f,s\right)-{\mu }_{2}\left(f,s\right)}{\sqrt{\frac{{\sigma }_{3}^{2}\left(f,s\right)+{\sigma }_{2}^{2}\left(f,s\right)}{2}}}$$

to measure how well a classifier based on the LLR discrimination statistic would be expected to distinguish the trisomy scenario from the euploid scenario. Notice that the sensitivity index d’ for normal distributions entirely determines the receiver operating characteristic curve and can be related to the area under the curve (AUC) via

$$d{^{\prime}}=\sqrt{2}{\Phi }^{-1}\left(AUC\right)$$

where Φ is the cumulative distribution function of the normal distribution.

To estimate what pairs of fetal DNA fraction and number of switch errors (f, s) provide comparable power to distinguishing the two scenarios, we estimate the sensitivity index d’(f, s) for fetal DNA fraction f in maternal plasma varying from 1.0% to 7.0% and for the number of switch errors s in the maternal homologs varying from 0 to 100 (Fig. 2c). The code used is publicly available.

### SNP-based targeted sequencing of DNA from maternal plasma

Two blood samples were drawn from the pregnant mother in two 10 ml Streck tubes at 11 weeks GA and the cfDNA was isolated from maternal plasma samples, amplified, and analyzed using the Natera Panorama v3 test^13^. The protocol includes a set of pooled primers targeting 13,926 distinct genetic loci, including 1351 SNPs on the 22q11.2 region to detect fetal 22q11.2 microdeletion^56^ and 277 Y-chromosome loci^73^ to infer sex and sex chromosome aneuploidies. Target SNPs have at least a 10% population minor allele frequency to ensure that a sufficient fraction would be heterozygous in any given patient^62^. Sequence data revealed that approximately three-quarters of targeted SNPs are present in HapMap^74^ and, cross-referencing with data from the 1000 Genomes Project^53^, the vast majority of SNPs have a mean worldwide minor-allele frequency greater than 35% with approximately 45% expected to be heterozygous in any given individual, similar to other strategies used to select SNPs that maximize heterozygosity^75^. Following amplification, libraries were run on the Illumina NextSeq 500 platform (Illumina, Inc., San Diego, CA)^56^ with 50 base pairs single-end sequence reads over three separate sequencing runs, likely because samples with ≤ 7% fetal DNA fraction are re-sequenced at a higher depth^19^. Read throughput for the three runs was 6.2, 7.1, and 16.5 million reads, for a total of 29.8 million reads.

### Massively parallel shotgun sequencing of DNA from saliva

High coverage MPSS data for the parents of the trisomy 18 fetus were available through Dante Labs^76^. Data were generated on the BGISEQ-500 platform, in paired ends 100 bp reads. Read throughput for the pregnant mother was 67.7, 76.0, 80.9, 58.7, 57.6, 49.3, 125.4, and 71.6 million read pairs, for a total of 587 million read pairs. Read throughput for the father of the fetus was 81.3, 89.3, 63.4, 64.8, 45.9, 83.7, 65.6, and 71.2 million read pairs, for a total of 565 million read pairs.

### Massively parallel shotgun sequencing of DNA from maternal plasma

Two blood samples were drawn in two 10 ml Streck tubes at 15 weeks GA. Plasma was separated from fresh whole blood according to the Streck cfDNA BCT tube protocol:

- Centrifuge whole blood at 300×*g* for 20 min at room temperature
- Remove the upper plasma layer carefully and transfer to a new conical tube
- Centrifuge the plasma at 5000×*g* for 10 min
- Remove plasma from any pelleted debris, and proceed to the isolation of the cfDNA.

We then followed the instructions provided in the QIAamp Circulating Nucleic Acid Kit (Qiagen Cat# 55114) to isolate the cfDNA according to the amount of plasma that has been collected. Multiple tubes of blood from a single patient can be processed on one single Qiagen kit column in order to concentrate the DNA while maintaining a low elution volume (important for library construction). We quantified the cfDNA prior to library construction on an Agilent TapeStation system using a high sensitivity D5000 ScreenTape.

Sequencing libraries were generated from the extracted cfDNA using the ThruPLEX Plasma-seq kit (Takara bio) following the instructions in the kit.

Note on library clean up: we have found that after library generation there is often a large quantity of high molecular weight library fragments that were produced from undesired DNA (library sizes 800–1200 bp). The library molecules generated from the desired cfDNA, which are around 300 bp in size (DNA insert + adapters), were enriched for by adding a “double cleanup” cleaning step using AMPure beads (Beckman Coulter), after first performing the 1:1 bead to sample post-amplification cleanup as described in the ThruPLEX protocol.

The double cleanup involved first adding 0.6X AMPure beads to the sample, incubating for 5 min, placing the sample on a magnetic rack, and collecting the supernatant. A volume of AMPure beads was again added to the supernatant to get to a 1:1 sample to bead ratio, incubating for 5 min, placing on a magnetic rack, and this time discarding the supernatant. The magnetic beads were washed twice with 80% ethanol and the library fragments were eluted from the beads and analyzed on the Agilent TapeStation d5000 screen tape for sequencing.

Libraries were run on the Illumina NextSeq 500 platform with a high output, 300 cycle kit, with 159 base pairs paired-end sequence reads, to maximize the odds of overlapping an informative polymorphic locus. Read throughput from the four lanes was 63.9, 65.4, 62.4, and 63.5 million read pairs, for a total of 255 million read pairs.

### Microarray-based genotyping of DNA from saliva

Microarray genotype data for three of the grandparents of the fetus were available through 23andMe^77^, AncestryDNA^78^, and FamilyTreeDNA^79^. We downloaded the genotype calls aligned against the GRCh37 human genome reference from the company’s respective websites and we converted the genotype calls to VCF using BCFtools convert^80^. We then lifted over genotypes coordinates to the GRCh38 human genome reference using the liftOver tool^81^, making sure to reverse complement alleles for SNPs whose coordinates flipped strands. Markers that failed to lift over were discarded.

### Sequence alignment and processing

Sequence reads were aligned against the GRCh38 human genome reference using bwa mem^82^. Aligned sequence reads were further processed with the MarkDuplicate Picard tool^83^ and base pair qualities were recalibrated using version 4.1.3.0 of the GATK Base Quality Score Recalibration walker according to GATK best practices^84^. Genotypes for DNA from saliva were called using version 4.1.3.0 of the GATK HaplotypeCaller walker^85^. Allelic depths for sequence fragments, rather than for sequence reads, were measured using version 4.0.12.0 of the GATK Mutect2 walker^86^, as at the time of the writing of this manuscript both the GATK HaplotypeCaller walker and newer versions of the GATK Mutect2 walker are unable to correctly annotate the allelic depth from overlapping sequence read pairs which occur frequently in MPSS data from maternal plasma due to the short size of cfDNA molecules^43^.

### Chromosomal phasing

Parental genotypes were pre-processed with BCFtools^80^ and phased using Eagle^41^, using the 1000 Genomes Project haplotype reference panel^53^ lifted over to the GRCh38 human genome reference. Chromosomal phase for the parents of the fetus was further improved using microarray genotype data from the grandparents of the fetus using the BCFtools trio-phase plugin part of the MoChA software^87^. Briefly, the trio-phase plugin determines the chromosomal phase at heterozygous sites for which at least one of the parental genotypes is determined as homozygous and then propagates this information at nearby heterozygous sites previously phased with Eagle for which the parental genotypes are either heterozygous or missing.

### Estimation of fetal DNA fraction

We estimated fetal DNA fraction from allelic ratios over heterozygous loci homozygous for opposite alleles in the parents^43^, that is, maternal homozygous loci for which the genotype of the fetus could be deterministically predicted as heterozygous. Given an autosome i and given p_i_ the number of sequenced reads with fetal-specific alleles and q_i_ the number of sequenced reads with alleles shared by the fetus and the mother across all such loci from autosome i, the fetal DNA fraction for that autosome i is estimated as:

$${f}_{i}=\frac{2{p}_{i}}{{p}_{i}+{q}_{i}}$$

These values were observed to be highly consistent across autosomes (Table 1) as previously reported^43^. To avoid potential biases due to aneuploid chromosomes, for each maternal plasma sample we estimated the fetal DNA fraction f as the median, rather than the mean, of the fetal DNA fractions f_i_ across the autosomes.

### Estimation of overdispersion

Although an initial commercial offer of the SNP-based method claimed to model allelic read counts as binomial random variables^15^, failure to properly model the overdispersion can cause artificially inflated LLR discrimination statistics as SPH segments from trisomy scenarios can erroneously fit the additional variance. Later iterations of the method introduce the use of beta-binomials to address this issue^63^, although this requires an additional parameter to be fit from the data. Measuring overdispersion is important when a large number of sequence fragments are sampled from each polymorphic locus with fragments that might originate from the same DNA molecule due to PCR amplification.

As the sequence data were generated from maternal plasma that included DNA from a male fetus, we used allelic read counts over chromosome X from the SNP-based test for which we do not expect additional variation due to inherited paternal homologs. We fit the intraclass correlation parameter ρ to maximize the product of beta-binomial likelihoods for the allelic read counts of the mother’s maternal alleles over SNP loci heterozygous for the mother along chromosome X restricting to loci covered by more than 200 sequence fragments, with allelic fractions for both alleles within 0.4 and 0.6 to exclude potential outliers due to small copy number variants, and further excluding regions Xp22.31, Xp22.32, and Xp22.33, as these regions have a different maternal inheritance state than the rest of chromosome X (Fig. 5c). The value ρ = 0.000936 was the value that best fit the allelic read counts from the SNP-based test and we rounded this value to ρ = 0.001, an estimate in agreement with independent modeling performed by a different group^66^. We caution that outlier loci can inflate the ρ parameter when fit as described, as the tails of a more overdispersed beta-binomial distribution can have much higher relative likelihoods than the tail of a less overdispersed beta-binomial distribution. We further fit the overdispersion parameter α to maximize the product of negative-binomial likelihoods for the total allelic read counts. The value α = 0.0697 was the best fit, and we rounded this value to α = 0.07 in our simulations. Notice that this value was not used in the computation of the LLR discrimination statistics, as the total allelic read counts are not used in the likelihood computations for the LLR.

### Maximum likelihood computations for empirical data

To model the expected allelic fractions from empirical data we first estimate, given both the maternal and the paternal inheritance states and both the maternal and paternal genotypes at a locus, what the expected fetal genotype at the locus is. Polymorphic loci likelihoods for each chromosome were computed as beta-binomial likelihoods P(k | n, π, ρ) with k the number of A alleles, n the total number of A and B alleles, π the expected fraction of A alleles in maternal plasma as a function of the estimated fetal DNA fraction f, maternal genotype, and fetal genotype, and ρ = 0.001 the intraclass correlation. For the model not using chromosomal phase, likelihoods P(k | n, π, ρ) and P(n-k | n, π, ρ) are averaged to obtain a combined likelihood that does not depend on chromosomal phase. To speed up likelihood computations across very many polymorphic loci, precomputed tables can be generated (Fig. S1).

Notice that in this model we assume the availability of both maternal and paternal homologs resolved with chromosomal phase, but a similar model could be designed where the paternal homologs inherited by the fetus are instead modeled through imputation using an external haplotype reference panel subsequent to inferring the fetal genotypes at loci where the mother is homozygous^44^.

We excluded from the likelihood computations indels, variants that failed variant quality score recalibration^84^, variants missing from the 1000 Genome Project haplotype reference panel^53^, and, due to bugs in the assembly graph determination in both the GATK HaplotypeCaller walker^85^ and the GATK Mutect2 walker^86^ at the time of the writing of this manuscript, variants that in the parents were less than 20 base pairs from each other. To avoid over-counting evidence at consecutive variants that might be covered by the same sequence fragments, we filtered variants to make sure a distance of at least 100 base pairs was always present between consecutive variants. For each chromosome, log_10_ likelihood ratio discrimination statistics were computed similar to what done for simulated allelic read counts. The code used is publicly available as a BCFtools plugin.

## Supplementary Information

Supplementary Figures.

## Data Availability

The data is freely available through the Personal Genome Project: https://my.pgp-hms.org/profile/hu058D3E sequence data from the pregnant mother https://my.pgp-hms.org/profile/huC1F919 sequence data from the father of the fetus.

## Code availability

https://github.com/freeseek/blueberry code to reproduce all analyses.
