# Supplementary material for: Chromosomal phase improves aneuploidy detection in non-invasive prenatal testing at low fetal DNA fractions
Source: Sci Rep. 2022 Jul 14;12:12025. doi: 10.1038/s41598-022-14049-5 (PMC9283487; doi:10.1038/s41598-022-14049-5)
Supplement: Supplementary file 1 — Supplementary Figures. [file 41598_2022_14049_MOESM1_ESM.pdf]

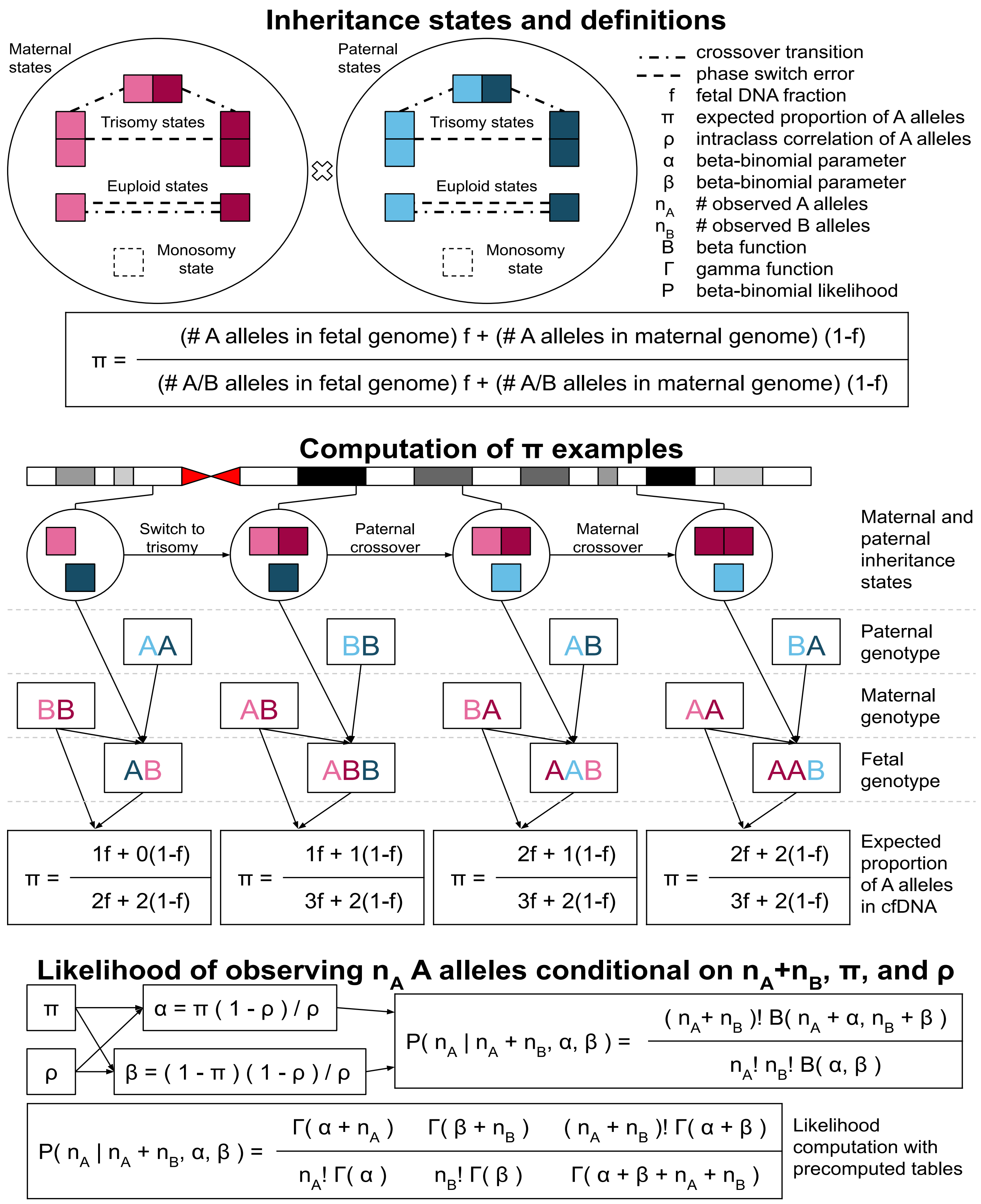

Figure S1. Description of the model used to compute $\log_{10}$ likelihood ratio (LLR) discrimination statistics. Graphical representation of how likelihoods at polymorphic markers are computed starting from a pair of parental inheritance states, a pair of phased parental genotypes, and an observation of n_A A alleles and n_B B alleles from the cfDNA at the marker's locus.

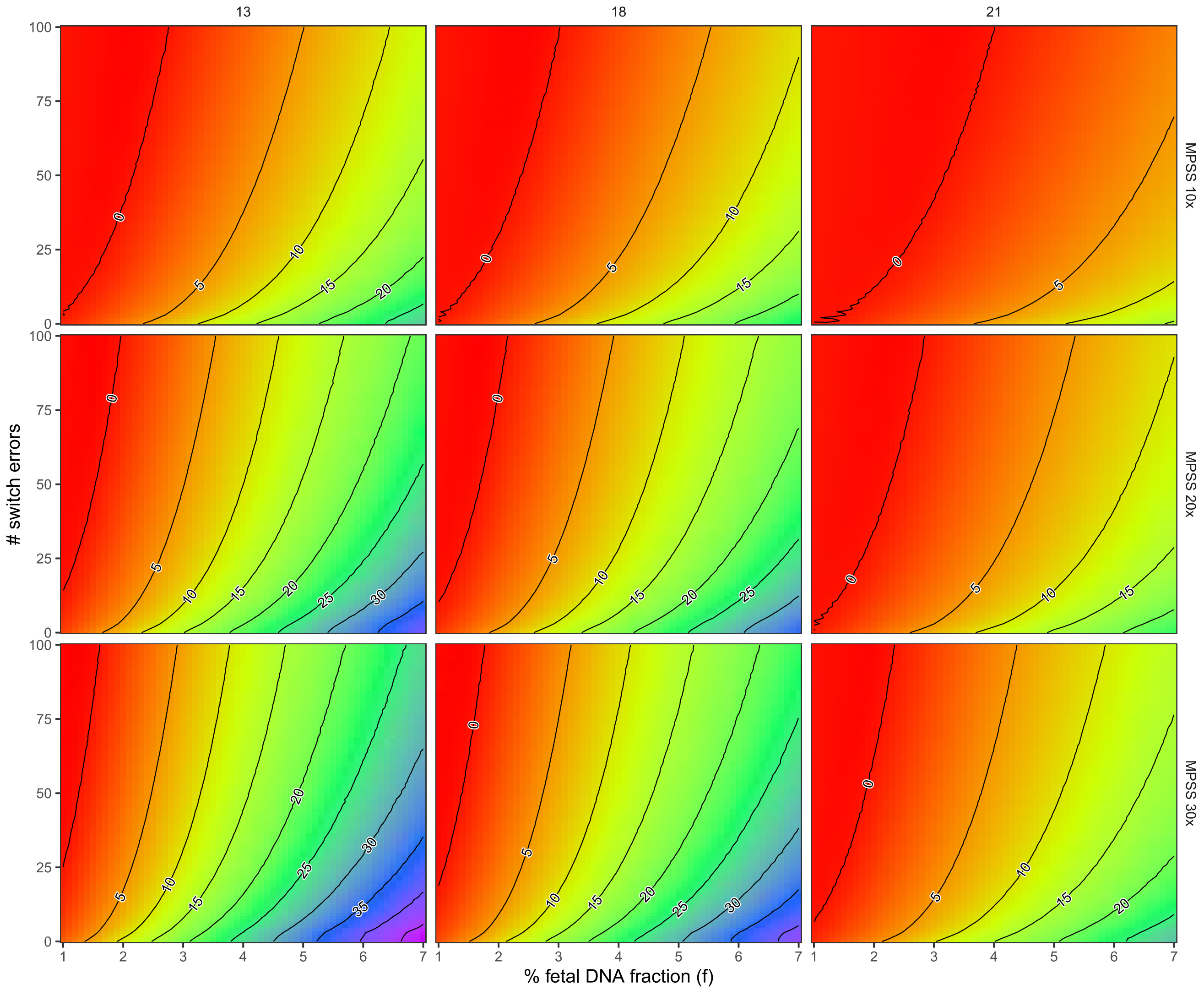

Figure S2. Simulation of sensitivity index d' for $\log_{10}$ likelihood ratio (LLR) discrimination statistics to detect trisomies. Sensitivity index d' between LLR discrimination statistics for allelic read counts simulated by trisomy scenarios and those simulated by euploid scenarios as a function of fetal DNA fraction and the number of switch errors with an average sampling of 10, 20, or 30 sequence fragments per locus for chromosomes 13, 18, and 21, simulated with, respectively, 52,609 41,585, and 19,878 heterozygous sites. Contour lines follow parameters sets with the same sensitivity index d', indicating scenarios with approximately equivalent power to distinguish euploid and trisomy scenarios.

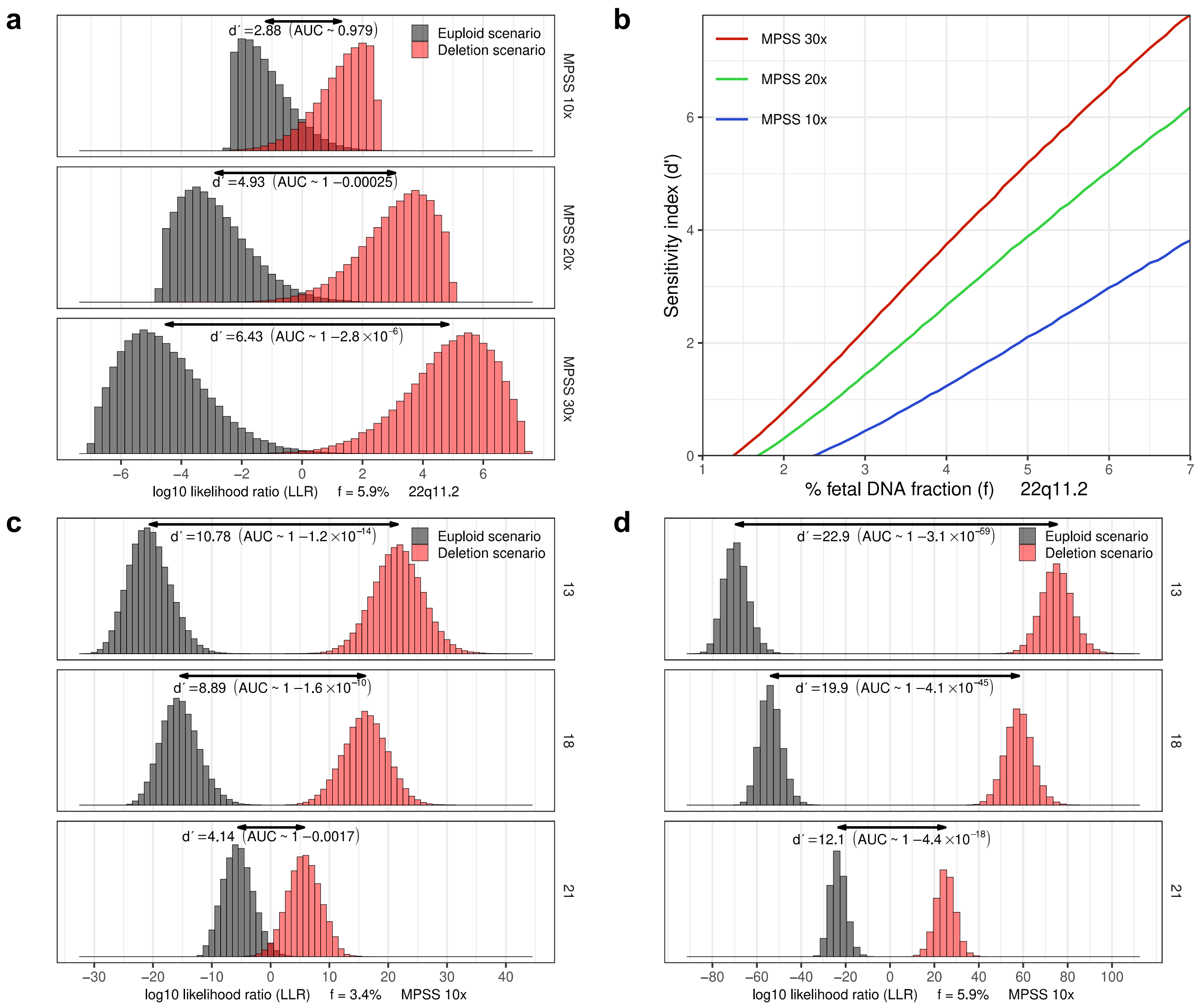

Figure S3. Simulation of $\log_{10}$ likelihood ratio (LLR) discrimination statistics for 22q11.2 microdeletion syndrome. a, **c**, **d** Simulated $\log_{10}$ likelihood ratio (LLR) discrimination statistics from simulations with no phase switch errors, a fetal DNA fraction specified as f=5.9%, an average sampling of either 10, 20, or 30 sequence fragments at either 1,340, 52,609, 41,585, and 19,878 loci heterozygous for the mother for, respectively, regions 22q11.2, chromosome 13, chromosome 18, or chromosome 21 for both euploid and microdeletion scenarios of maternal origin. Sensitivity index d' between the LLR discrimination statistics for the two scenarios is displayed together with the AUC estimated as if the two LLR discrimination statistics were normally distributed. **b**, Sensitivity index d' between LLR discrimination statistics for allelic read counts simulated by microdeletion end euploid scenarios as a function of fetal DNA fraction with an average sampling of 10, 20, or 30 sequence fragments showing that power at low fetal DNA fraction (<4%) is limited with low coverage MPSS.

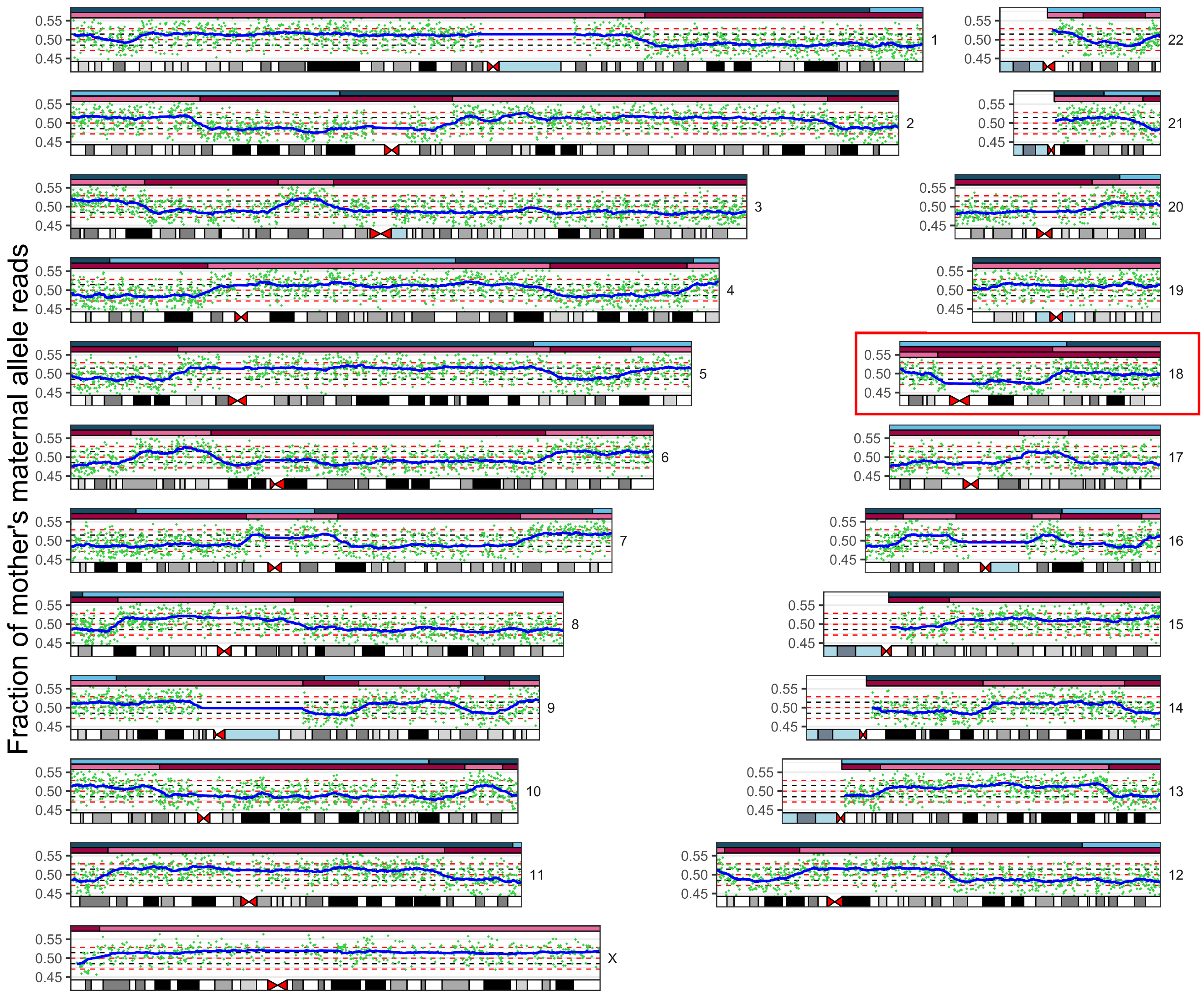

Figure S4. Massively parallel shotgun sequencing data with chromosomal phase. Graphical representation of sequence data at loci consistent with heterozygous genotypes for the mother estimated from MPSS of maternal plasma at 15 weeks GA with a fetal DNA fraction estimated as f=5.9%. Each green point represents the proportion of mother's maternal alleles across 100 consecutive loci heterozygous for the mother. The two black dotted bars represent the expected proportions of mother's maternal alleles in the case of a euploid fetus, and the three red dotted bars represent the expected proportions in the case of a fetus with trisomy of maternal origin. The top bars represent the inferred inherited homologs of the fetus, with magenta, red, cyan, and blue colors representing, respectively, mother's maternal, mother's paternal, father's maternal, and father's paternal homologs. Chromosome 18, with three fetal homologs inferred, is highlighted in red. It is important to note that the algorithm to infer the inherited homologs takes also into account information about the father's homologs and allelic read counts at loci consistent with homozygous genotype for the mother which are not displayed in this figure and that the fetal homologs inherited from the father further add to the sampling noise at loci heterozygous for the mother.
